# Data set on mortality of national basketball association (NBA) players

**DOI:** 10.1016/j.dib.2022.108615

**Published:** 2022-09-17

**Authors:** Jose A. Martínez, Klaus Langohr, Julián Felipo, Luciano Consuegra, Martí Casals

**Affiliations:** a Department of Business Economics, Universidad Polite´cnica de Cartagena, Cartagena 30201, Spain; b Department of Statistics and Operations Research, Universitat Politècnica de Catalunya/BarcelonaTECH, Barcelona 08034, Spain; c Newsroom, Basketball Departament, Mundo Deportivo, Barcelona 08036, Spain; d Unidad de Hemodinámica Cardíaca, Servicio de Cardiología, Hospital Universitario de Santa Lucia, Cartagena, Murcia, Spain; e Sport and Physical Activity Studies Centre (CEEAF), University of Vic–Central University of Catalonia (UVic-UCC), Vic 08500, Spain; f National Institute of Physical Education of Catalonia (INEFC), University of Barcelona, Barcelona, Spain

**Keywords:** Basketball, Mortality, NBA, Sports statistics, Survival analysis, Standardized mortality ratio

## Abstract

In this work, we present a data set on the survival times and mortality rates of all 4374 professional basketball players who participated in the National Basketball Association (NBA) from its inception in 1946 until July 2019 [1]. It contains the data of 412 active and 3962 former players. The data were recorded from different internet sources and include information on each player's position, ethnicity, handedness, ages at NBA debut and career end, height, weight, or number of NBA games. The results of the analysis of a previous data set with the same variables of all NBA players from 1946 to 2015 were recently published by Martinez *et al.* in 2019 [2]. The information provided in the data set can be useful to better understand the mortality risk among NBA players.

## Specifications Table

**Table:** 

Subject	Health and medical sciences
Specific subject area	Sport Sciences, Therapy and Medicine
Type of data	Tables and figures
How data were acquired	We employed the database of the retrospective cohort study of Martinez *et al.* (2019) [2], adding four more years of data (from April 2015 to July 2019). All data were recorded from different internet sources, especially from the webpages of Sports Reference, LLC [3] and Newspapers.com [4].
Data format	Raw
Description of data collection	On one hand, we entirely checked the original database of Martinez *et al.* (2019) [2] in order to detect possible codification errors or changes in the information provided by the internet sources reviewed. On the other hand, the database was updated with the information of all new NBA players since 2015. In addition, the vital status by July 2019 of all NBA players was checked.
Data source location	Raw data sources: https://www.newspapers.com, https://www.basketball-reference.com, https://www.nba.com, https://www.fiba.com, https://www.apbr.org, https://www.nytimes.com, https://www.latimes.com, https://www.espn.com.
Data accessibility	Repository name: GitHub Data identification number: 10.5281/zenodo.6883911 Direct URL to data: https://github.com/marticasals/Reproducibility_Mortality_NBA_Players/tree/main/Data
Related research article	J. A. Martinez, K. Langohr, J. Felipo, M. Casals, 2019. Mortality of NBA Players: Risk Factors and Comparison with the General US Population, Applied Sciences. 9, 500. https://www.mdpi.com/2076-3417/9/3/500.

## Value of the Data

• This data set is an extensive compilation of the survival and current vital states of all former and active NBA players from the inception of the NBA in 1946 until 2019. In addition, it provides information on the characteristics of the NBA players such as position, ethnicity, ages at debut and career end, height, weight, or number of NBA games.
• The information provided in the data set can be useful for practitioners, medical staff, and NBA-related associations such as the National Basketball Athletic Trainers Association, the National Basketball Players Association, or the National Basketball Retired Players Association to better understand the mortality risk of NBA players.
• These data might be used for other studies on the death risk of NBA players. Furthermore, it might be interesting to compare the data to those on the mortality of professional sportsmen and women from other professional leagues.
• Following the International Olympic Committee's (IOC) ruling on protection and pro- motion of health among athletes, it is well known that the first step in the public health approach for preventing diseases, mortality, and their impact is to collect accurate data and surveillance systems.
• Reproducible and reliable mortality data on professional NBA players are needed to in- form strategic health care service decision-making. These data can represent a dynamic register of mortality useful to improve accuracy and continue adding the data over seasons to continue doing research.
• Respecting confidentiality, ethics, data privacy, and analytics in professional sports, these data can help mostly scientists, the National Basketball Association (NBA), IOC research centers, and the players themselves to jointly promote a health strategy based on surveillance systems with better data records.

## 1 Data Description

### 1.1 Raw and processed data

The data set on the mortality of National Basketball Association's players can be downloaded from both Github (https://github.com/marticasals/Reproducibility_Mortality_NBA_Players/tree/main/Data and Zenodo [1]. The raw data, which were all collected by the authors (see Section 2.1), are contained in the first sheet of the EXCEL file NBA raw data2019.xlsx within the folder Data in either repository. A short description of all the variables collected is provided in the second sheet of this EXCEL file.

The file NBA raw data2019.xlsx contains the following information of all the 4374 professional basketball players that played at least one NBA game until the end of the 2018/2019 season: name; date of birth; present status (former or active player); position; origin (USA/ not from the USA); ethnicity; NBA debut; first and last NBA seasons; vital status by 31 July 2019; date of death in the case of players that died before 31 July 2019; age at death or 31 July 2019; age at NBA debut; weight (in kilograms); height (in centimeters); number of NBA games; and handedness.

In addition, the folder Data contains the file MortalityUSA2000to2018.xlsx with the age-specific mortality rates among the African-American and White populations in the USA from 2000 through 2018. These rates were used to compute the standardized mortality ratios of observed to expected mortality rates among NBA players (see Section 2.2). The source of these mortality rates are the web pages of the Centers for Disease Control and Prevention [5].

The R script DataImportToR.R in the same folder was employed to read the data from both EXCEL files in R and to create the R workspace NBAdata2019.RData, which contains the data frames that were used for the analysis of the data. Both the R workspace and the R script of the data analysis (RCode DiB Martinez Langohr Casals EtAl.R) are included in the folder Analysis DiB in either repository.

### 1.2 Tables and figures

Table 1 presents the characteristics of all 4374 professional basketball players that are included in the data set according to their status by July 2019 —412 were active players and 3962 were former players—, and the characteristics of those 864 (19.8%) NBA players who had died before 31 July 2019. The mean height and weight of the NBA players were 198 cm (standard deviation: 9.3 cm) and 95.1 kg (12 kg), respectively. A total of 2592 players (59.3%) were African-American, 1613 (36.9%) were White, 13 (0.3%) were Asian, 137 (3.1%) were Mixed and in 19 cases (0.4%), ethnicity was unknown. The distribution of the players’ positions is: 11.4% were centers, 12.4% were forward–centers, 28.7% were forwards, 12.5% were forward–guards, and 35% were guards. In addition, the average age at the NBA debut was 23.3 years (2.1 years), and the average age at the end of the NBA career of the former NBA players was 28.3 years (4.4 years).

**Table 1:** Descriptive analysis of active (by 31 July 2019) and former NBA players, and those players who died.

	Total∗	Active∗	Former∗	Players who died∗∗
	*N* = 4374	*N* = 412	*N* = 3962	*N* = 864
**Position**				
Center	498 (11.4%)	51 (12.4%)	447 (11.3%)	88 (19.7%)
Forward-Center	544 (12.4%)	39 (9.5%)	505 (12.7%)	178 (35.2%)
Forward	1256 (28.7%)	107 (26.0%)	1149 (29.0%)	214 (18.6%)
Guard-Forward	547 (12.5%)	60 (14.5%)	487 (12.3%)	149 (30.6%)
Guard	1529 (35.0%)	155 (37.6%)	1374 (34.7%)	235 (17.1%)
**Ethnicity**				
African-American	2592 (59.3%)	284 (68.9%)	2308 (58.3%)	225 (9.7%)
White	1613 (36.9%)	83 (20.1%)	1530 (38.6%)	630 (41.2%)
Asian	13 (0.3%)	1 (0.3%)	12 (0.3%)	0
Mixed	137 (3.1%)	40 (9.7%)	97 (2.4%)	5 (5.2%)
Unknown	19 (0.4%)	4 (1.0%)	15 (0.4%)	4 (26.7%)
**Origin**				
USA	3925 (89.7%)	317 (76.9%)	3608 (91.1%)	848 (23.5%)
Non-USA	449 (10.3%)	95 (23.1%)	354 (8.9%)	16 (4.5%)
**Left handed**				
No	4108 (93.9%)	372 (90.3%)	3736 (94.3%)	832 (22.3)
Yes	266 (6.1%)	40 (9.7%)	226 (5.7%)	32 (14.2)
**Age at debut** (in years)∗∗∗	23.3 (2.1)	21.7 (1.8)	23.5 (2.1)	24.5 (2.6)
**Age at end of career** (years)∗∗∗	–	–	28.3 (4.4)	27.7 (3.7)
**Height** (cm)∗∗∗	198 (9.3)	200 (8.7)	198 (9.3)	194 (9.0)
**Weight** (kg)∗∗∗	95.1 (12.0)	98.4 (11.0)	94.7 (12.0)	90.1 (10.1)
**Number of NBA games**∗∗∗	260 (302)	193 (188)	267 (310)	188 (239)

∗ Percentages represent proportions among all, active, and former players, respectively.

∗∗ Percentages represent proportions among former players of the same category.

∗∗∗ Summary statistics of numeric variables are mean and standard deviation.

**Fig. 1:**
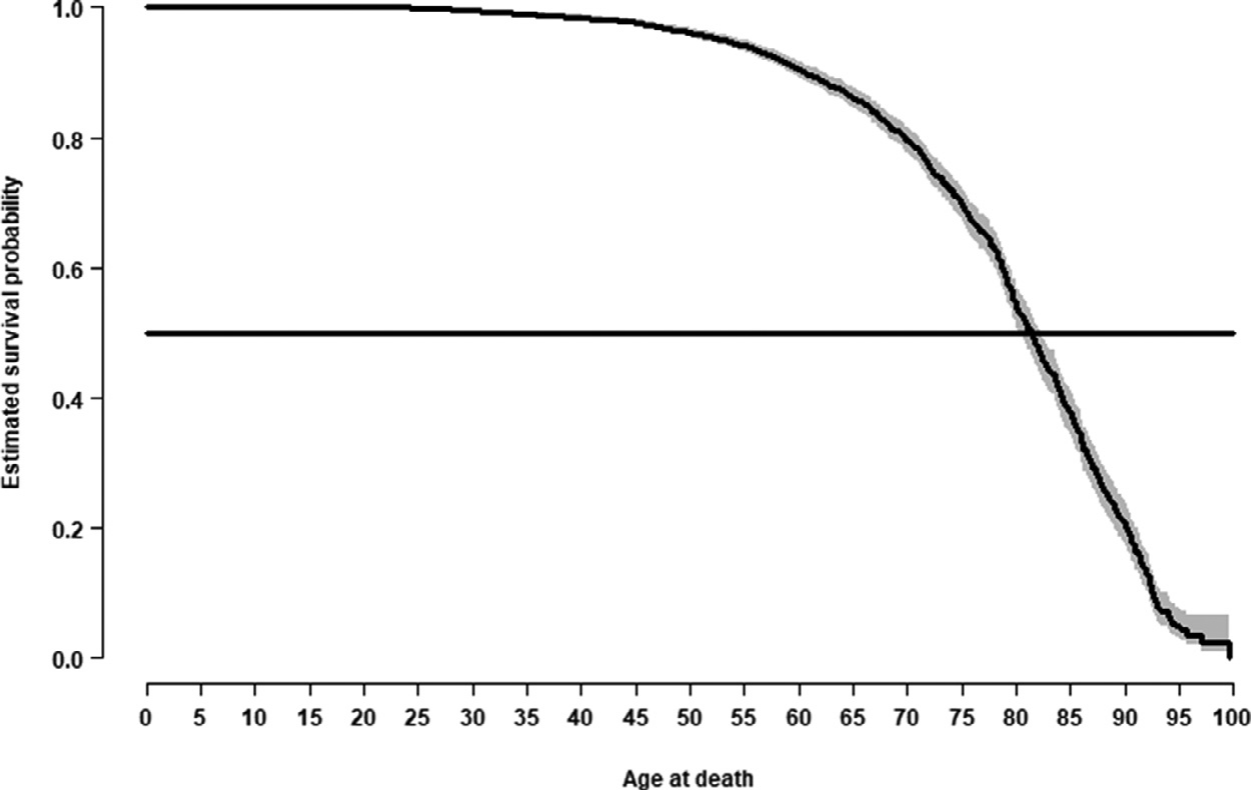
Estimated survival probabilities as a function of age of NBA players. The shaded area represents the corresponding 95% confidence bands and the horizontal line represents the survival probability of 0.5.

Concerning the age at death of NBA players, the times until either death or 31 July 2019 of all 4374 NBA players were used for the nonparametric estimation of the survival function, which is shown in Fig. 1. The youngest NBA player to die was Bryce Dejean-Jones, who was fatally shot in 2016 at the age of 23 years, and the oldest former NBA player to die was Ben Goldfadden, who died in 2013 at the age of 99 years. The estimated median age at death was 81.4 years.

The information provided in the data set allows the fit of regression models to analyze the survival times of NBA players and to identify factors possibly related to these survival times.

The proportional hazards model of Cox shown in Table 2 is the updated version of the model presented in Martinez *et al.* [2]. According to this model, the height of the NBA players is related to survival time and the positive sign of the estimated parameter indicates that the instantaneous death risk increases with height. In addition, the comparison of African-American players to White players reveals a higher instantaneous death risk among the former with an estimated adjusted hazard ratio of 1.4. The negative sign of the parameter estimate corresponding to the first NBA season reflects the fact that life expectancy has increased in recent decades. Notice that for this analysis, we have only considered the data of the African-American and White NBA players.

**Table 2:** Fit of Cox's proportional hazard model to analyze factors associated with mortality among NBA players during the period 1946–2019. The response variable is age at death.

	Estimate	SE*	HR (95% CI)⁎⁎	p-value
**Height**	0.021	0.004	1.021 (1.012-1.03)	<0.001
**Ethnicity (African-Americans vs White)**	0.326	0.099	1.386 (1.141 – 1.683)	0.001
**Year of first NBA season**	-0.024	0.004	0.976 (0.968 – 0.984)	<0.001

⁎ Standard error.

⁎⁎ Hazard ratio (95%-Confidence interval).

**Fig. 2:**
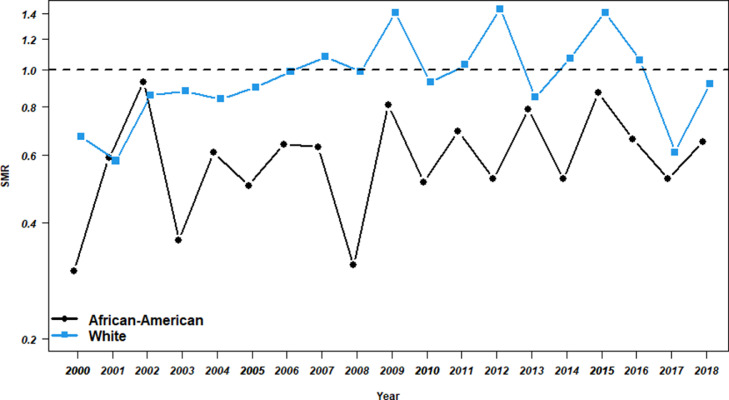
Standardized mortality ratios of observed to expected mortality among African-American and White NBA players. Computation of the expected mortality is based on the mortality rates from the respective general US populations.

Fig. 2 shows the standardized mortality ratios (SMRs) among African-American and White NBA players from 2000 through 2018. In most of the years analyzed, the SMRs were smaller than 1, that is, the mortality rates among the NBA players were lower than the ones among the respective general populations. In the case of the White NBA players, the differences are not very large and some SMRs are, even, greater than 1. By contrast, the SMRs of the African-American NBA players are always smaller than one with most values close to 0.6; these values reflect a greater difference in mortality with respect to the general African-American population.

## 2 Experimental Design, Materials and Methods

### 2.1 Data collection

In the following lines, we summarize the steps we followed to build up both the original data set, which was analyzed and presented in Martinez *et al.*, 2019 [2], and the updated data set, in order to make the understanding of the complete process easier:

*a) Original research*

• We recorded the information on all 3985 NBA players (3504 former and 481 by then active players) who had played in the NBA from its inception in 1946 to April 2015. The data source used was https://www.basketball-reference.com.
• Individuals who only had participated in the American Basketball Association (ABA) League were excluded.
• All the biophysical and technical variables that were available and could be considered relevant for the study were registered: place of birth, playing position, career games played, career minutes played, date of NBA debut, and last season played. In addition, weight, date of birth, and date of death (in the case of deceased players) were added to the database by two of the authors and three independent coders, who received prior training for that purpose. Another variable (handedness) was registered manually by two of the authors without any recorded mismatches.
• Two additional variables (ethnicity and height) were also manually recorded by following a different validation procedure: ethnicity was recorded by two independent coders previously trained, who categorized players in African-American, White, Asian, and Mixed. Dubious cases were discussed by all the authors and the final classification was reached by consensus.

On the other hand, the obtained records of height were compared with the information available on the https://www.nba.com website, in order to detect disparities. We detected 43 different values of height from our initial data and the values on https://www.nba.com were used in the case of these mismatches. In addition, we checked the database of the International Basketball Federation (FIBA) to compare heights of NBA players who had played any FIBA tournament, identifying 238 players with slight variations. As there was no way to decide which of the sources was most reliable we regressed FIBA heights on NBA heights for the 238 players identified and observed a statistically significant regression coefficient (0.964) with an R-squared of 0.953. Using these parameters, we estimated the heights of the remaining 3757 NBA players as a proxy of their FIBA height. Therefore, we had two different versions of the height variable.

• We finally recorded information on the 687 former NBA players who had died in the considered period of analysis. We employed different data sources for that purpose, mainly basketball networks (APBR), digital newspapers (New York Times, LA Times), and sports websites (ESPN).

*b) Updated data set*

• We extended the end of the analysis from April 2015 to July 2019. Therefore, the complete period of analysis is from 1946 to July 2019.
• We used the same data sources as in the original study, but we also included a focused search in the historical database of https://www.newspapers.com, which was also employed in other similar studies of mortality and sports; e.g., Tanaka *et al.*, 2021 [6]).
• The number of players considered grew from 3985 (3505 former and 481 active) to 4374 (3962 former and 412 active).
• We did not use trained coders to record some variables as we did in the original research. All the new information was added manually by the authors, who checked independently the variables of ethnicity and handedness. Regarding height, we employed the source of https://www.basketball-reference.com.
• The number of deceased players increased from 687 to 864.
• We considered several issues to build the new database and to improve the data quality: for example, there were 24 players whose date of abandoning the NBA changed because of a hiatus in their participation in the league before and after April 2015. We also considered the 386 new players who achieved their debut in the NBA after 15 April 2015, and three players that were missed in the original database and who debuted in March and April 2015. Of the 174 new deaths taken into account in this new analysis, 131 were deaths from May 2015 to July 2019, and 43 were deaths that were not previously identified on the web site of Sports Reference, LLC [3] in the study of Martinez et al (2019), but that were correctly identified in this new work after multiple checking for each player in different sources.

Although this study is one of the few that shows public and validated data as one of the first NBA player mortality information systems from different sources, we must not forget that it relies on publicly available data, which is noisy in nature. We have taken the approach to embrace the noise of the data set and accept it is an unavoidable reality of dealing with public player mortality data.

### 2.2 Statistical analysis

For the descriptive analysis of all former and active NBA players presented in Table 1, we computed absolute and relative frequencies in the case of categorical variables, and the means and standard deviations of the numeric variables.

The response variable used to analyze the NBA players’ survival times was age at death. This analysis was based on the data of all 4374 NBA players treating the survival times of those players alive by 31 July 2019 as right-censored data. To correctly analyze the data and avoid too optimistic estimations of survival probabilities, all survival times were treated as left-truncated at the age of the corresponding NBA debut. We estimated the survival probabilities and the median age at death with the Kaplan-Meier method [7] and conducted a multivariate analysis using Cox's proportional hazards model [8]. The potential predictors considered for the analysis were the players’ physical characteristics and NBA career-related variables. Furthermore, the model was adjusted for the year of the players’ first NBA season to account for the fact that life expectancy had improved throughout the past decades. The effect size measure of the Cox model is the hazard ratio, i.e., the ratio of the instantaneous risk functions, which is assumed to be constant over time. For the model fit presented in Table 2, only African-American and White players were considered, and the proportional hazard assumption of the model was graphically checked using the Schoenfeld residuals [9].

For the comparison of the mortality of African-American and White NBA players to that of the general African-American and White US populations, respectively, we computed standardized mortality ratios (SMRs) for the years 2000 through 2018. This measure is the ratio of the observed to the expected number of deaths in the study population, where the yearly expected number of deaths was calculated based on the players’ ages on January 1, and the survival probabilities of the same year in the corresponding population.

All analyses were conducted using version 4.1.2 of the R statistical software (Vienna, Austria; https://www.r-project.org/). In particular, R packages survival [10] and *epitools* [11] were used to analyze the survival times and to compute the SMRs, respectively. The R code of the analyses carried out and the data are available in GitHub (https://github.com/marticasals/Reproducibility_Mortality_NBA_Players/tree/main/Analysis_DiB). This work fulfils the transparency and reproducibility of scientific research.

## Ethics Statement

We did not seek ethical approval for this work, as all information used and reported is freely available via online sources.

## CRediT Author Statement

**Jose A. Martinez:** Conceptualization, Methodology, Data curation, Investigation, Writing – original draft, review & editing, Supervision; **Klaus Langohr:** Conceptualization, Methodology, Data curation, Investigation, Writing – original draft, review & editing, Visualization, Software, Validation; **Julián Felipo:** Data curation, Investigation; **Luciano Consuegra:** Data curation, Investigation; **Martí Casals:** Conceptualization, Methodology, Data curation, Investigation, Writing – original draft, review & editing, Software, Validation.

## Declaration of Competing Interest

The authors declare that they have no known competing financial interests or personal relationships which have, or could be perceived to have, influenced the work reported in this article.

## Data Availability

Reproducibility Mortality NBA Players (Original data).
